# *Agave angustifolia* Haw. Leaves as a Potential Source of Bioactive Compounds: Extraction Optimization and Extract Characterization

**DOI:** 10.3390/molecules29051137

**Published:** 2024-03-03

**Authors:** Misael Bermúdez-Bazán, Mirna Estarrón-Espinosa, Gustavo Adolfo Castillo-Herrera, Antonio Escobedo-Reyes, Judith Esmeralda Urias-Silvas, Eugenia Lugo-Cervantes, Anne Gschaedler-Mathis

**Affiliations:** 1 Centro de Investigación y Asistencia en Tecnología y Diseño del Estado de Jalisco, A.C., Unidad de Tecnología Alimentaria, Camino Arenero 1227, El Bajío, Zapopan 45019, Jalisco, Mexico; bermude1@ualberta.ca (M.B.-B.); jurias@ciatej.mx (J.E.U.-S.); elugo@ciatej.mx (E.L.-C.); 2 Centro de Investigación y Asistencia en Tecnología y Diseño del Estado de Jalisco, A.C., Unidad de Servicios Analíticos y Metrológicos, Av. Normalistas No. 800, Guadalajara 44270, Jalisco, Mexico; aescobedo@ciatej.mx; 3 Centro de Investigación y Asistencia en Tecnología y Diseño del Estado de Jalisco, A.C., Unidad de Biotecnología Industrial, Camino Arenero 1227, El Bajío, Zapopan 45019, Jalisco, Mexico; agschaedler@ciatej.mx

**Keywords:** *Agave*, agro-wastes, bioactive compounds, extraction, optimization, vegetal extracts, mass spectrometry

## Abstract

The leaves of *Agave angustifolia* Haw. are the main agro-waste generated by the mezcal industry and are becoming an important source of bioactive compounds, such as phenolic compounds, that could be used in the food and pharmaceutical industries. Therefore, the extraction and identification of these phytochemicals would revalorize these leaf by-products. Herein, maceration and supercritical carbon dioxide (scCO_2_) extractions were optimized to maximize the phenolic and flavonoid contents and the antioxidant capacity of vegetal extracts of *A. angustifolia* Haw. In the maceration process, the optimal extraction condition was a water–ethanol mixture (63:37% *v*/*v*), which yielded a total phenolic and flavonoid content of 27.92 ± 0.90 mg EAG/g DL and 12.85 ± 0.53 µg QE/g DL, respectively, and an antioxidant capacity of 32.67 ± 0.91 (ABTS assay), 17.30 ± 0.36 (DPPH assay), and 13.92 ± 0.78 (FRAP assay) µM TE/g DL. Using supercritical extraction, the optimal conditions for polyphenol recovery were 60 °C, 320 bar, and 10% *v*/*v*. It was also observed that lower proportions of cosolvent decreased the polyphenol extraction more than pressure and temperature. In both optimized extracts, a total of 29 glycosylated flavonoid derivatives were identified using LC-ESI-QTof/MS. In addition, another eight novel compounds were identified in the supercritical extracts, showing the efficiency of the cosolvent for recovering new flavonoid derivatives.

## 1. Introduction

*Agave angustifolia* Haw. (*A. angustifolia* Haw.), also known as Maguey “Espadín”, is a plant from the *Agave* genus with a wide distribution that stretches from the southern United States to Ecuador [1]. In Mexico, *A. angustifolia* cores are mainly used to elaborate mezcal compared to other agave species. The state of Oaxaca is the main mezcal producer, contributing 91.32% of the national mezcal production in 2022 [2].

According to the Mexican Regulator Council of the Quality of Mezcal [2,3], the annual production of mezcal dramatically increased from 8.099 to 14.165 million liters from 2021 to 2022. However, the high demand for this beverage in the alcoholic beverage market has posed an environmental concern due to the generation of agro-wastes (i.e., leaves and bagasse) in the mezcal manufacturing process. Agave leaves represent ~50% of the total weight of the agave and contribute to the vegetal wastes [4]. To date, the annual amount of leaves discarded by the mezcal industry into the environment remains unknown.

It is known that the agave agro-wastes are potential sources of phytochemicals such as polyphenolic compounds [5,6]. Some studies have determined the phytochemical composition of different agave species using advanced analytical tools. For example, Maazoun et al. (2019) [7] identified various glycosylated flavonoid derivatives of quercetin, kaempferol, isorhamnetin, and ellagic acid in a methanolic extract from *Agave americana* leaves using HPLC-ESI/TOF-MS. Moreover, Morreeuw et al. (2021a) [8] identified glycosylated flavonoid derivatives of isorhamnetin, hesperidin, cyanidin, and delphinidin in ethanol–water (70:30 *v*/*v*) and methanol–water (60:40 *v*/*v*) extracts from *Agave lechuguilla* leaves using an ultrasound-assisted extraction process. The phenolic content and the quantitative content of the identified polyphenolic compounds were higher using an aqueous methanol mixture owing to the high polarity of organic polar solvents in aqueous mixtures [9]. More recently, Morreeuw et al. (2021b) [10] identified mono-, di-, and triglycosylated flavonoids of apigenin, isorhamnetin, kaempferol, quercetin, and myricetin in ethanolic extracts of *A. lechuguilla* using HPLC-MS. Regarding *A. angustifolia* Haw., a smaller number of studies have evaluated the polyphenolic composition of this species. El-Hawary et al. (2020) reported for the first time the occurrence of new phenolic acids in *A. angustifolia* var. *marginata*, such as fukiic acid and piscidic acid, and other flavonoids such as eucomol and dyhydroeucomin [11]. The presence of these flavonoids was associated with the immune-modulatory and anti-inflammatory activities of the vegetal extract [11]. Other studies investigated the antioxidant capacity of *A. angustifolia* foliar extracts, revealing high radical scavenging and reductant capacities [12]. Furthermore, vegetal extracts of *A. angustifolia* were employed to increase the shelf-life of a meat product, showing outstanding antibacterial, radical scavenging, and reductant activities owing to the high phenolic content [13]. Based on these studies, the leaves of *A. angustifolia* are a feasible source of polyphenolic compounds.

Although there are studies evaluating the composition and potential applications of polyphenolic compounds from the leaves of *A. angustifolia*, establishing optimal extraction conditions for these compounds without compromising their antioxidant properties has not been addressed. Supercritical fluid extraction (SFE) is a green extraction technology that separates analytes from a complex matrix (e.g., vegetal or food samples) based on their relative solubility [14]. Reportedly, SFE yields natural products with a high content of polyphenolic compounds using short extraction periods, making it a more efficient and less time-consuming process [15]. This is because a supercritical fluid exhibits gas-like viscosity and liquid properties, which means a high diffusivity, strong solvation power, and high density [16]. The unique properties of a supercritical fluid make it suitable for permeating porous matrices and rapidly diffusing into them, improving the mass transfer of the solutes from the extraction medium [17]. When it comes to extracting phenolic compounds, supercritical carbon dioxide (scCO_2_) is typically used, and the process is often assisted using a polar cosolvent to enhance the solvation power of the scCO_2_ [18]. Regardless of this, the required amount of cosolvent is often lower than that of conventional extraction technologies, which is another advantage of scCO_2_ extraction [15,19]. Additionally, the solvent capacity of scCO_2_ can be controlled by varying the pressure and temperature through modulating the solvent density and solvation power. In this way, the polarity of the extraction system can be controlled, enhancing the selectivity of the extraction process. To our knowledge, scCO_2_ extraction has rarely been used to obtain natural products from agave species. In fact, it has only been used in one study to recover natural products (saponins) from *Agave salmiana* bagasse [20]. Nevertheless, the employment of scCO_2_ extraction for the recovery of phenolic compounds from the leaves of *A. angustifolia* Haw. has not been addressed.

To overcome this problem, scCO_2_ extraction can be employed to obtain polyphenolic compounds from the leaves of *A. angustoflia* Haw. to add value to this agro-waste. To further guarantee the effectiveness of scCO_2_ extraction, suitable cosolvent and extraction conditions should be carefully established. Several studies have focused on the optimization of various extraction processes from different kinds of by-products from the food industry using surface response methodology (SRM) [21,22,23,24]. Furthermore, SRM allows us to analyze and process data from a response variable of interest that is influenced by different quantitative factors (independent variables) [25]. The main objective of SRM is to design an experiment that determines the specific values of the independent variables to elicit the desirable effect on the response variable. For SFE, the Box–Behnken design (BBD) is a suitable option for optimizing the extraction of polyphenolic compounds because it avoids conducting experiments in extreme conditions [26]. Moreover, BBD excludes the combination of the highest and lowest values of the independent variables in the design, and the number of total runs is lower than in other experimental designs, which allows us to save time and operation costs [26].

In this research, it was of primary concern to identify a suitable cosolvent before conducting the SFE process. In this sense, the first step of this research was to optimize the cosolvent mixture using a conventional maceration extraction process. Recent studies have shown that organic polar solvents (e.g., methanol, ethanol, acetone) mixed with water can substantially enhance the extraction of polyphenolic compounds owing to the greater polarity of polar organic solvents in aqueous mixtures [27,28]. One possible way to obtain a desirable solvent mixture that allows for high recovery of polyphenolic compounds is to use a simplex–centroid mixture design [29,30,31]. Therefore, the first aim of this study was to systematically optimize the proportions of the polar mixture components (acetone, ethanol, and water) using a mixture design. In the current study, this optimization was a crucial step in providing a suitable solvent mixture with high polyphenol recovery performance for use as the solvent in scCO_2_ extraction. Considering polyphenolic compounds′ radical scavenging properties and hydrogen/electron donation ability to suppress highly oxidative species [32], the antioxidant capacity of the resulting natural products (extracts) was chosen as the main response variable to optimize both processes. Therefore, the second aim of our study was to maximize not only the phenolic and flavonoid contents of the obtained extracts but also to increase the antioxidant capacity of the extracts from both extraction processes. Finally, it was hypothesized that scCO_2_ would allow us to obtain novel polyphenolic compounds despite using the same matrix. To address this hypothesis, the third aim of this research was to investigate the qualitative composition of the obtained extracts from both optimized processes. In this way, this research provides more knowledge related to the polyphenols of *A. angustifolia* Haw. and provides a process to add value to the vegetal agro-wastes.

## 2. Results

### 2.1. Model Fitting and Analysis of Variance of the Evaluated Response Variables in Maceration Extraction

All the data were analyzed by fitting a reduced cubic model in the mixture design. Tables S1 and S2 in the Supplementary Material show the ANOVA and model-fitting results of the reduced cubic model for total phenolic content (TPC), total flavonoid content (TFC), extraction yield (EY), and antioxidant capacity (AC). TPC, TFC, EY, and AC (ABTS, DPPH, FRAP assays) showed determination coefficient (R^2^) values ≥ 0.90, whereas the adjusted R^2^ values for all variables were ≥0.70. The statistical parameter for lack of fit did not show any statistical differences between all evaluated variables (Tables S1 and S2), corroborating the model prediction ability. Furthermore, the predicted R^2^ values ranged from 0.82 to 0.96 for all tested variables except for DPPH. The values of the adjusted and predicted determination coefficients indicated that the reduced cubic model was reliable in predicting each response variable [30]. To support this, a remarkable correlation was observed between the predicted and experimental values for all variables (Figure S2).

Regarding the analysis of variance, Tables S1 and S2 show the magnitude of the regression coefficients corresponding to each solvent and the *p* values. According to the results, individual solvents and their combinations showed significant differences, with the recovery of phenolic and flavonoid compounds, extraction yield, and antioxidant capacity dependent on the solvent type and proportion. The high values of the regression coefficients for each response variable represent an effective fit of the model [33].

### 2.2. Determination of the Total Phenolic Content and Antioxidant Activity in Extracts from the Maceration Process

**Figure 1:**
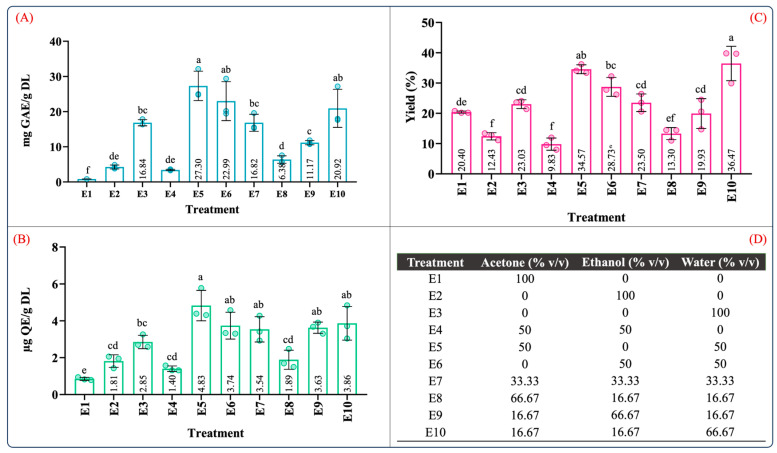
Quantification of (**A**) total phenolic content, (**B**) total flavonoid content, (**C**) extraction yield, and (**D**) mixture design treatments of the obtained extracts from leaves of *Agave angustifolia* Haw. using maceration extraction. Results are presented as data mean ± standard deviation (n = 3) and analyzed by one-way ANOVA (*p* ≤ 0.05) with a significance level (α) of 95%. Distinct letters indicate different mean and statistically significant differences (*p* ≤ 0.05).

Figure 1 depicts the TPC, TFC, and yield of the ten extraction treatments in the mixture design for the maceration process. Overall, from the results depicted in Figure 1, the phenolic and flavonoid contents ranged from 0.84 to 27.30 mg GAE/g DL and 0.85 to 4.83 µg QE/g DL, and the yield ranged between 9.83 and 36.47%. These values were consistent with the range of the phenolic and flavonoid contents reported in *A. angustifolia* leaves [12,13]. Furthermore, for individual extraction solvents, the highest phenolic content (16.84 ± 1.66 mg GAE/g DL), flavonoid content (2.85 ± 0.36 µg QE/g DL), and extraction yield (23.03 ± 1.44, g dry extract/g DL) was produced using the aqueous extract (E3), followed by ethanol (E2) and acetone (E1). However, the aqueous binary mixtures dramatically increased the recovery of the phenolic and flavonoid contents as well as the extraction yield (Figure 1A–C).

Similarly, the total phenolic and flavonoid contents of the ethanol aqueous mixture (E6) were nearly six-fold and two-fold higher than that of the ethanol extract (E2). These significant differences can be attributed to an increase in the solvation power of the solvent system provided by the water in the binary aqueous mixtures [27], favoring and facilitating the extraction of water-soluble and organic-soluble molecules [34].

**Figure 2:**
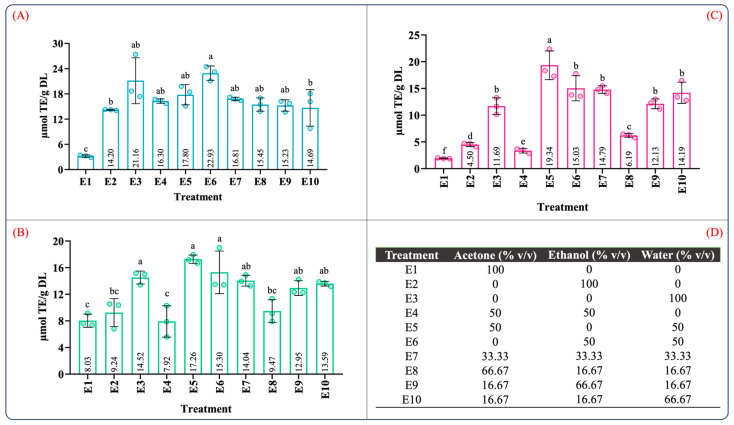
Determination of the antioxidant capacity of extracts from leaves of *Agave angustifolia* Haw. using maceration extraction. (**A**) ABTS assay, (**B**) DPPH assay, (**C**) FRAP assay, and (**D**) mixture design treatments of the obtained extracts from leaves of *Agave angustifolia* Haw. using maceration extraction. Results are presented as data mean ± standard deviation (n = 3) and analyzed by one-way ANOVA (*p* ≤ 0.05) with a significance level (α) of 95%. Distinct letters indicate different mean and statistically significant differences (*p* ≤ 0.05).

Table S2 summarizes the ANOVA results for the antioxidant capacity evaluated by ABTS, DPPH, and FRAP assays. According to Table S2, the type of solvent employed in the maceration process and the mixture composition significantly influenced the antioxidant capacity (*p* < 0.05). Figure 2 illustrates the effect of the individual solvents and mixture composition on the antioxidant capacity. As can be seen in Figure 2, the antioxidant capacity was 3.20–22.93, 7.92–17.26, and 1.92–19.34 µM TE/g DL in the ABTS, DPPH, and FRAP assays, respectively. In the individual solvent systems, the aqueous extract (E3) showed the highest antioxidant capacity in the ABTS, DPPH, and FRAP assays (Figure 2A–C). As expected, the antioxidant capacity using the aqueous organic binary mixtures (E5: water–acetone 50:50 (*v*/*v*); E6: water–ethanol 50:50 (*v*/*v*)) was notably higher than that of the individual solvents. Interestingly, the extracts using ternary blends with a high proportion of water and ethanol (E10 and E9) showed antioxidant capacity values similar to those of the aqueous organic binary mixtures (Figure 2A–C). These results showed that extracts obtained using a mixture with a higher proportion of polar solvents have a better antioxidant capacity; this behavior was also observed by Jdaini et al. (2023) and Ameer et al. (2017) [31,35]. In general, the antioxidant capacity (Figure 2) and the phenolic and flavonoid contents (Figure 1) results presented similar behaviors, suggesting that polyphenol content and the antioxidant capacity are directly proportional, which was confirmed through the Pearson correlation coefficient analysis (*r*^2^).

The correlation results between the polyphenolic content and antioxidant capacity are summarized in Table 1. As expected, a very strong significant correlation was found between TPC with DPPH (*r*^2^ = 0.824; *p* < 0.05) and FRAP (*r*^2^ = 0.963; *p* < 0.05) as well as TFC with FRAP (*r*^2^ = 0.880; *p* < 0.05). Similarly, the correlation between TFC and DPPH (*r*^2^ = 0.775; *p* < 0.05) was strong and significant. In contrast with the strong correlations obtained for FRAP and DPPH with TPC and TFC, the correlations between ABTS with TPC (*r*^2^ = 0.610; *p* < 0.05) and TFC (*r*^2^ = 0.518; *p* < 0.05) were significant but moderate. Moreover, a quite strong correlation was observed between DPPH and FRAP (*r*^2^ = 0.845; *p* < 0.05). These findings indicated that higher phenolic and flavonoid contents are associated with a greater antioxidant capacity. In addition, this result confirmed that the phenolic and flavonoid contents in the extracts are directly related to the reducing capacity of the phenolic compounds contained in the extracts, which reflects their antioxidant activity [36].

The correlation between the FRAP and DPPH assay results indicates that both assays follow the same reaction mechanism, which is an electron transfer from the antioxidant to the oxidant [37]. These results are consistent with the study reported by Barriada-Bernal et al. (2014) [38], where a strong correlation was found between flavonoids and their Fe^+3^ ion-reducing capacity in extracts from *Agave durangensis*. Contrary to our results, a study reported by Puente-Garza et al. (2017) [39] did not find a correlation between the phenolic and flavonol contents and the antioxidant capacity; instead, it was found that saponins were significantly correlated with the antioxidant capacity of extracts from *A. salmiana*. In our study, the phenolic and flavonoid contents displayed moderate correlation coefficient values with the ABTS assay results. This may be attributed to other hydrophilic or hydrophobic compounds other than phenolic compounds since the ABTS assay can evaluate the antioxidant capacity of polar and non-polar metabolites [40,41].

### 2.3. Optimization of Maceration Extraction Conditions

**Figure 3:**
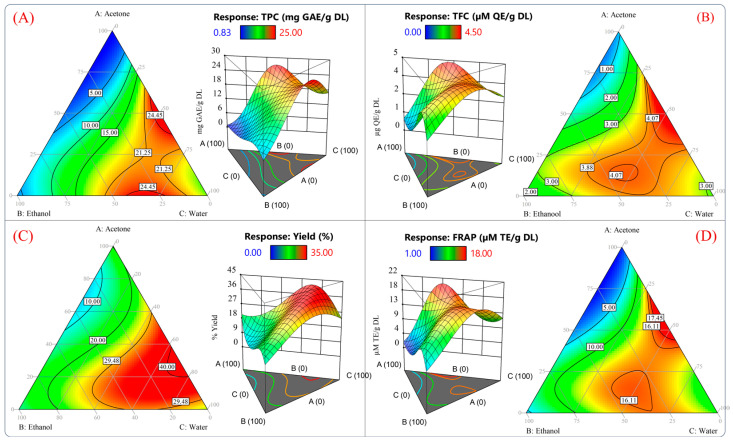
Surface response and contour plots of the dependent variables of the reduced cubic model. (**A**) Total phenolic content, (**B**) total flavonoid compounds, (**C**) extraction yield, and (**D**) antioxidant capacity measured by FRAP assay.

Figure 3 plots the contours and surface response of the predicted values from the model for TPC, TFC, extraction yield, and antioxidant capacity measured by the FRAP assay. According to Figure 3A–D, it would be possible to reach values of 24.45 mg GAE/g DL, 4.07 µg QE/g DL, 40%, and 16.11 µM TE/g DL using the theoretical conditions of 46.27/4.08/49.64 *v*/*v*/*v* % acetone/water/ethanol. Furthermore, a high antioxidant capacity and high yield of polyphenols could be obtained using 2.99/30.86/66.13 *v*/*v*/*v* % acetone/ethanol/water. Using these proportions, the model predicted values of 21.25 mg GAE/g DL, 3.88 µg QE/g DL, 29.48%, and 16.11 µM TE/g DL for each variable (Figure 3A–D).

These results suggested that the best proportion to yield an extract with a high antioxidant capacity and phenolic compound content would be 46.27%/4.08%/49.64% and 25.08%/20.11%/54.70% *v*/*v*/*v* acetone/ethanol/water, respectively. In Figure 3, it should be noted that the predicted values for all the response variables between the mixtures 46.27/4.08/49.64 *v*/*v*/*v* % and 2.99/30.86/66.13 *v*/*v*/*v* % acetone/ethanol/water were similar. Therefore, the optimization of the solvent proportions was conducted by reducing the acetone and maximizing the antioxidant capacity of an aqueous ethanolic mixture. The previously mentioned criteria were adopted because the acetone extract showed poor antioxidant activity and inefficient recovery of phenolic compounds (Figure 1 and Figure 2). Furthermore, ethanol and water are suitable solvents for pharmaceutical and food applications [42].

#### Validation of the Theoretical Optimized Conditions of the Maceration Extraction 

Once the criterion of the optimization of the maceration process was established, the theoretical solutions predicted by the model were analyzed using the desirability function in all the evaluated responses (see Figure S1). Figure S1 in the Supplementary Material shows the predicted responses as a function of the desirability by following the previously described optimization criteria. Figure S2 shows that all the predicted responses under the extraction conditions of 63:37 *v*/*v* ethanol–water produced an extract with a high antioxidant capacity, TPC, TFC, and extraction yield with desirability of 0.88. This binary aqueous mixture was experimentally tested to validate the optimized conditions predicted by the model. Table 2 shows that the predicted and experimental values of all evaluated responses using the optimized conditions (63:37 *v*/*v* water–ethanol) were in good agreement, confirming that the postulated model was accurate and reliable. These results demonstrated that the 63:37 (*v*/*v*) water–ethanol mixture was the best solvent for producing an extract with high phenolic and flavonoid contents and antioxidant capacity. Therefore, this hydroalcoholic mixture was used as the cosolvent to optimize an scCO_2_ process. 

### 2.4. Model Fitting and Analysis of Variance of the Evaluated Response Variables in scCO_2_ Extraction

To optimize a supercritical carbon dioxide extraction process, all the dependent variables were analyzed using a quadratic regression model, except the ABTS data, which were analyzed using a reduced cubic model. Tables S3 and S4 summarize the ANOVA and model-fitting results. All the dependent variables in the postulated models were significantly different (*p* < 0.05). Regarding the statistical parameters, the determination coefficient values (R^2^) were ≥0.9 for all tested variables, and there were no statistical differences in the lack of fit (*p* > 0.05). In addition, the predicted and adjusted R^2^ values were ≥0.80. These results suggest that the postulated models had an acceptable fit and seemed reliable in making predictions for each response variable. The extraction yield was analyzed once per extraction condition and was not included in the surface response analysis.

Tables S3 and S4 summarize the regression coefficient and *p* values for the evaluated dependent variables, namely temperature (A), pressure (B), and cosolvent concentration (C). The TPC, TFC, and antioxidant capacity were influenced by the extraction conditions and the linear interactions between the independent variables, such as pressure–cosolvent (B × C) and temperature–cosolvent (A × C) interactions. The pressure–temperature interaction (A × B) was only significant for the ABTS variable. In addition, their quadratic effect (A^2^; B^2^; C^2^) also displayed significant differences (Tables S3 and S4). Since the interactions and quadratic effects of the independent variables had specific effects on the response variables, their behavior will be discussed in the next section. 

#### 2.4.1. Effect of Temperature, Pressure, and Cosolvent on the Total Phenolic Content, Flavonoid Content, and Extraction Yield 

**Figure 4:**
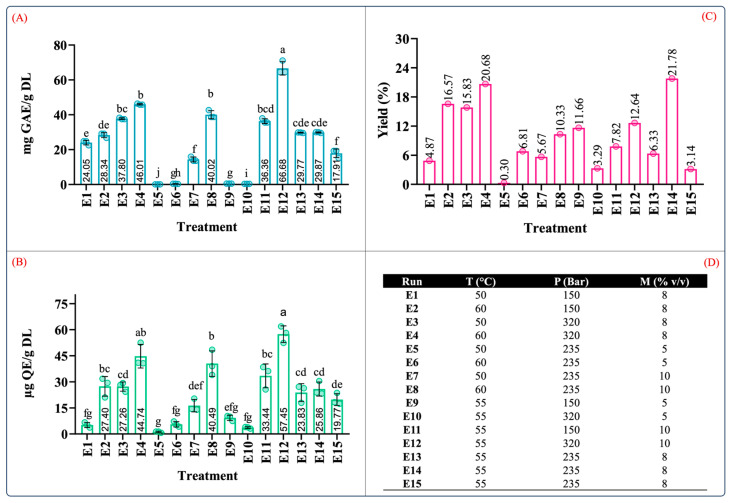
Quantification of phenolic content in extracts from leaves of *Agave angustifolia* Haw. using supercritical fluid extraction. (**A**) Total phenolic content, (**B**) total flavonoid content, (**C**) extraction yield, and (**D**) Box-Behnken design treatments of the obtained extracts from the leaves of *A. angustifiolia* Haw. using scCO_2_ extraction. Results are presented as data mean ± standard deviation (n = 3) and analyzed by one-way ANOVA (*p* ≤ 0.05) with a significance level (α) of 95%. Distinct letters indicate different mean and statistically significant differences (*p* ≤ 0.05).

Figure 4 depicts the results of the total phenolic and flavonoid contents of the extract produced by supercritical fluid extraction tests with their corresponding yields. From Figure 4A–C, it can be observed that the total phenolic and flavonoid contents and extraction yield ranged from 0.10 to 66.68 mg GAE/g DL, 1.14 to 57.45 µg QE/g DL, and 0.30 to 21.78%, respectively. As indicated by the variance analyses, the recovery of these compounds was affected by the extraction conditions as well as the interactions between the independent variables.

The conditions that produced extracts with the highest phenolic content (in descending order) were E12 (55 °C, 320 bar, 10% *v*/*v*), E4 (60 °C, 320 bar, 8% *v*/*v*), and E8 (60 °C, 235 bar, 10% *v*/*v*) at 66.68 ± 8.35, 46.01 ± 2.32, and 40.01 ± 2.32 mg GAE/g DL, respectively (Figure 4A). Similarly, this trend was also observed in the TFC results. The conditions that produced extracts with the highest TFC (in descending order) were E12 (57.45 ± 4.77 µg QE/g DL), E4 (44.4 ± 6.75 µg QE/g DL), and E8 (40.49 ± 7.34 µg QE/g DL) (Figure 4B). It should be noted that the conditions that resulted in the largest values of TPC and TFC were those conducted with moderate levels of pressure, temperature, and cosolvent concentrations. 

**Figure 5:**
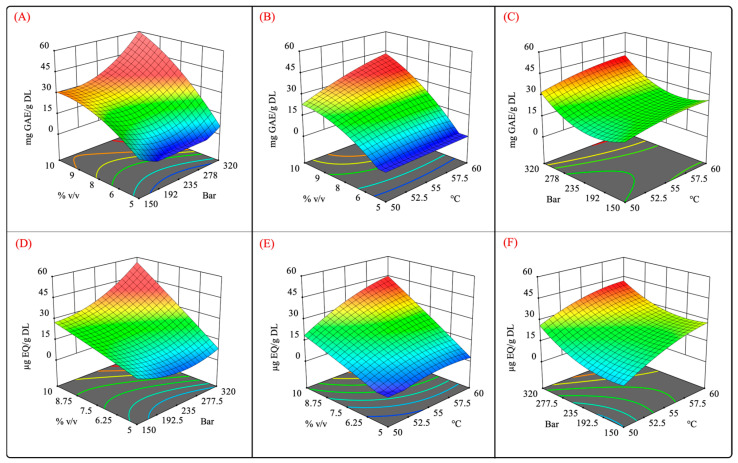
Surface response plots for independent variables vs. phenolic and flavonoid contents. (**A**,**D**) pressure–%cosolvent; (**B**,**E**) temperature–%cosolvent; and (**C**,**F**) pressure–temperature interactions.

Figure 5 shows the surface response plots of the interaction of the independent variables for the scCO_2_ extraction. It was observed that high-pressure extraction allowed for a better recovery of phenolic and flavonoid compounds (Figure 5A,D). Moreover, the pressure also exhibited a quadratic effect on TPC and TFC. Different studies that aimed to extract phenolic compounds from different vegetal matrices reported that the employment of high pressure enhanced the CO_2_ density and solvation power, further improving the dissolution of phenolic compounds [43,44,45,46]. Similarly, high levels of the cosolvent promoted a high recovery of phenolic and flavonoid compounds. From these results, it can be concluded that there is a synergistic effect between pressure and the cosolvent owing to the high density of scCO_2_ and greater polarity of the extraction system using a polar cosolvent mixture (Figure 5A,D). In addition, the use of aqueous organic mixtures has been shown to increase the solubility of polyphenolic compounds by weakening their hydrogen bonds [47]. 

It has been reported that the addition of hydroalcoholic solvents into the supercritical extraction process can influence the selectivity and solubility of extractable compounds through van der Waals forces, hydrogen bonding, and other complex interactions [47]. Furthermore, hydroalcoholic cosolvents can improve the permeability of the sample tissue, improving the mass transfer, molecular diffusion, and solubilization of various classes of hydrophilic compounds [48,49,50,51].

As mentioned previously, the cosolvent–temperature interaction was significant (*p* < 0.05). Figure 5B,E show that the cosolvent positively impacted the recovery of phenolic and flavonoid compounds at high temperatures. This result suggests that the solubility of these compounds is increased at certain temperatures, which is similar to the results reported by Santos-Zea et al. (2019) [20]. 

No significant differences were observed regarding pressure–temperature interactions, but at constant cosolvent concentrations, high pressures and temperatures enhanced the recovery of phenolic compounds. This result suggests that both factors positively impact the TPC and TFC. High pressure levels at a constant cosolvent concentration intensify the solvation power of scCO_2_ [52], whereas elevated temperatures boost the mass transfer rate [53]. However, it was also reported that high temperatures may also result in a decrease in the density of scCO_2_ [54], diminishing its solvation power. 

Here, it is important to highlight the paramount importance of the addition of the optimized hydroalcoholic mixture because it allowed us to enhance the efficiency of phenolic and flavonoid compound extraction. Additionally, it addressed the negative effect of temperature on the extraction system. Various studies reported that the employment of hydroalcoholic mixtures could boost the extraction efficiency for polyphenols using supercritical fluid extraction [18,55,56,57].

To the best of our knowledge, there is only one study focused on extracting secondary metabolites from the *Agave* genus using supercritical fluids [20]. However, it was conducted to obtain saponins from the bagasse of *A. salmiana*. To the best of our knowledge, this is the first research focused on optimizing the extraction of phenolic compounds from *A. angustifolia* leaves using scCO_2_ extraction with an aqueous cosolvent.

#### 2.4.2. Effect of Temperature, Pressure, and Cosolvent on the Antioxidant Capacity

**Figure 6:**
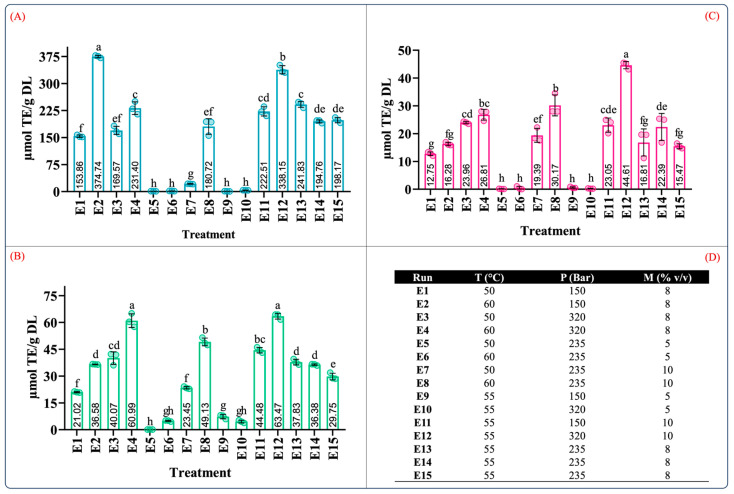
Determination of the antioxidant capacity from leaves of *Agave angustifolia* Haw. in the supercritical extraction. (**A**) ABTS assay, (**B**) DPPH assay, (**C**) FRAP assay, and and (**D**) Box-Behnken design treatments of the obtained extracts from the leaves of *A. angustifiolia* Haw. using scCO_2_ extraction. Results are presented as data mean ± standard deviation (n = 3) and analyzed by one-way ANOVA (*p ≤* 0.05) with a significance level (α) of 95%. Distinct letters indicate different mean and statistically significant differences (*p ≤* 0.05).

Figure 6 presents the antioxidant capacity of the extracts from all the scCO_2_ extraction experiments measured by the ABTS, DPPH, and FRAP assays. Overall, the antioxidant capacity, as measured by the ABTS, DPPH, and FRAP assays, ranged from 0.80 to 374.74, 0.12 to 63.47, and 0.10 to 44.61 µmol TE/g DL, respectively (Figure 6A–C). As expected, the antioxidant capacity was influenced by the operational parameters of the process. The conditions that produced extracts with the highest antioxidant capacity (in descending order) were E12 (55 °C, 320 bar, 8% *v*/*v*), E4 (60 °C, 320 bar, 8% *v*/*v*), and E8 (60 °C, 235 bar, 10% *v*/*v*), according to the DPPH and FRAP assay results (Figure 6B,C). Despite the large differences observed between the ABTS assay values and the values from the DPPH and FRAP assays, a similar trend was observed (Figure 6A–C). An inverse behavior was observed for condition E2 (60 °C, 320 bar, 8% *v*/*v*), which displayed the highest antioxidant capacity in the ABTS assay. In this condition, the high temperature may have had a positive impact on the antioxidant capacity since high temperature can enhance the mass transfer properties of the solutes despite decreasing the solubility of scCO_2_ [54].

**Figure 7:**
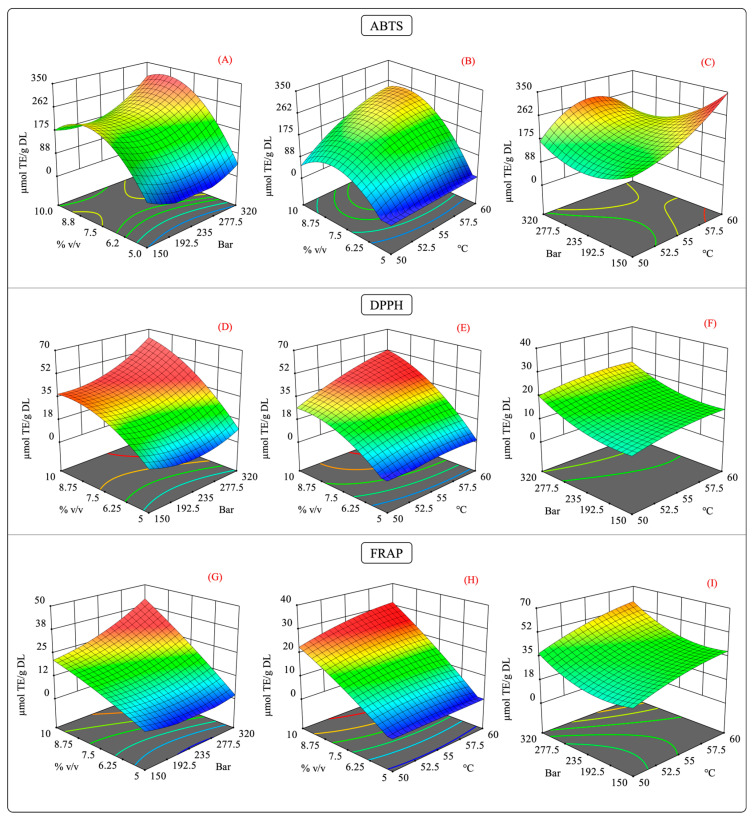
Surface response contour plots of the combined effects of the independent variables on the antioxidant capacity in the scCO_2_ extraction. (**A**,**D**,**G**) interaction effect of pressure-%cosolvent on the antioxidant capacity of ABTS, DPPH, and FRAP; (**B**,**E**,**H**) interaction effect of temperature-%cosolvent on the antioxidant capacity of ABTS, DPPH, and FRAP; and (**C**,**F**,**I**) interaction effect of pressure-temperature on the antioxidant capacity of ABTS, DPPH, and FRAP.

As previously mentioned, the interaction of the independent variables of the antioxidant capacity behaved in a specific manner. Figure 7 illustrates the surface response of the interaction of antioxidant capacity measured by the ABTS, DPPH, and FRAP assays. In the pressure–cosolvent interaction, there was a substantial increase in the antioxidant capacity at constant temperature levels (Figure 7A,D,G). In this case, the factor that mainly influenced the antioxidant capacity was the high-pressure levels, which agreed with the larger values of the quadratic regression coefficient for these variables (Table S4).

The impact of the temperature–cosolvent interaction was positive because, at constant pressure, the high cosolvent concentration increased the antioxidant capacity at certain temperatures. Moreover, a quadratic effect of the temperature and cosolvent on the ABTS and DPPH assay results was observed (Figure 7B and Figure 7E, respectively). This behavior agrees with the significant differences in the quadratic terms for the temperature and cosolvent (Table S4). In the FRAP assay results, no significant differences were observed in the quadratic terms for the temperature and cosolvent; instead, the quadratic term for pressure was significant. This result suggests that at a constant pressure, the temperature–cosolvent interaction variable had an important influence on the antioxidant capacity.

The effect of the pressure–temperature interaction on the antioxidant capacity showed statistically significant differences in the ABTS assay results but not in those of the DPPH and FRAP assays. For the antioxidant activity, this interaction was more complex since an additional term was required to explain the variability of the model (A^2^×) (Table S4). 

From Figure 7C, it should be noted that operating the extraction system at high pressure and temperature levels negatively impacted the antioxidant capacity. This behavior may be explained by a competing effect for the solubility of the scCO_2_ caused by the high levels of these factors [58]. As discussed previously, high temperatures can decrease the density of scCO_2_, reducing its solvation power. The contrary effect is produced by increasing the pressure in the extraction system [59]. Therefore, it is possible that such increases in both factors counteracted the enhancement in the solvation power of scCO_2_, which in turn could have slowed the diffusion of the solutes into the extraction medium [60,61]. As in maceration extraction, for scCO_2_ extraction, the experiments showed that extracts with a higher antioxidant capacity had high phenolic and flavonoid contents, which suggests a possible correlation between these variables. 

In Table 3, a good correlation was confirmed between the antioxidant capacity (measured by the three assays) and TPC and TFC.

As previously discussed, this result indicates that the antioxidant capacity of the extracts obtained by scCO_2_ is strongly associated with the recovered polyphenols from leaves of *A. angustifolia* Haw.

#### 2.4.3. Optimization and Validity of the Theoretical Extraction Conditions for scCO_2_ Extraction

From the previous results, an obvious conclusion was that the cosolvent concentration was the factor that substantially influenced the recovery of polyphenolic compounds and, consequently, the antioxidant activity, which was accepted because, as mentioned before, scCO_2_ shows a non-polar behavior, and it could be modified to have a less non-polar property by the addition of a cosolvent (which has a greater effect compared to high pressure). To support this conclusion, it was observed that cosolvent concentrations >5% were required to achieve the optimal response of the dependent variables. In addition, a high cosolvent concentration compensated for the negative effect caused by the competing effects of high temperature and pressure. Based on this evidence, it was hypothesized that the process could be conducted at high temperatures and pressures. 

**Figure 8:**
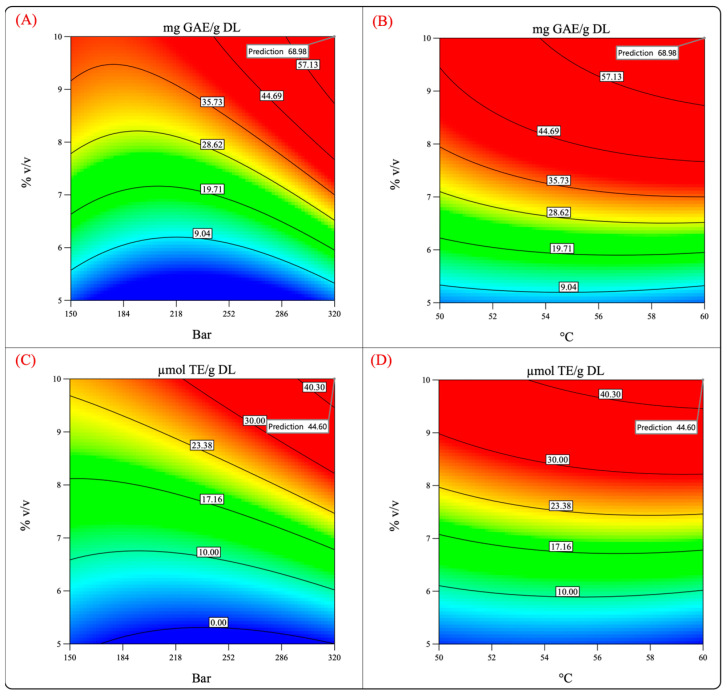
Predicted values of the total phenolic content and antioxidant activity (evaluated by FRAP assay). (**A**,**C**) Pressure–cosolvent interaction; (**B**,**D**) temperature–cosolvent interaction.

Figure 8 shows the surface response and contour plots of the antioxidant capacity and phenolic content. It can be observed that by performing the process at 320 bar, 60 °C, and 10% cosolvent, the phenolic content and the antioxidant capacity reached high values. Therefore, the extraction was performed under such conditions to test the validity of the model and the previously mentioned hypothesis.

Table 4 summarizes the values predicted by the model and the experimental values obtained by experimentation. The experimental values were quite different from those predicted by the model, which indicates that the validation of the theoretical conditions was not accomplished.

After the optimal extraction was performed, significant changes in the *A. angustifolia* leaves powder were observed, which may indicate that factors such as swelling, compaction, and some channeling could have affected the efficient flow of the cosolvent (a significant factor in the extraction process) inside the matrix, decreasing the phenolic recovery. These factors were not considered in the model, making it difficult to simulate the results. The cosolvent absorption in fibrous vegetal samples and swelling within the extraction vessel produce a compacted bed. This issue was also reported by Santos-Zea et al. (2019) [20]. Moreover, the compacted bed formed in the vegetal matrix may increase the surface tension and viscosity of the water in the cosolvent, hindering the extraction process [18,52]. For this reason, sample swelling and compaction of the raw material should be considered before extracting polyphenols from powdered agave leaves in future studies.

### 2.5. LC-ESI-QTof/MS Characterization of Polyphenols in the Extracts Obtained Using Optimized Maceration and scCO_2_ Extraction Conditions

Despite not validating the optimal conditions predicted by our model for scCO_2_ extractions, it was of great interest to us to investigate whether such experimental conditions assured a suitable recovery of phenolic compounds using both extraction methods. Therefore, we generated a qualitative profile of the phenolic compounds in the vegetal extracts obtained by maceration and scCO_2_ extraction (SFE extract).

Table 5 shows the tentatively identified compounds in both extracts based on their *m*/*z* from MS spectra in negative and positive ionization modes ([M−H]^−^/[M+H]^+^) using a Waters^®^ Xevo G2-XS ESI-Qtof Mass Spectrometer. The compounds with a mass error ≤ 10 ppm were selected for characterization [62].

A total of 24 different compounds in the vegetal hydroalcoholic extracts of *A. angustifolia* leaves obtained by maceration and scCO_2_ extraction were characterized. The characterized compounds included 14 glycosylated flavonoids, 7 phenolic acids, 1 glycosylated coumarin, 1 glycosylated terpene, and 1 glycosylated xanthone. 

In the hydroalcoholic extracts obtained by maceration and scCO_2_ extraction, three phenolic subclasses were detected: hydroxybenzoic acids, cinnamic acids, and stilbenes. The cinnamic acid derivatives were detected in both extracts and tentatively identified as glycosyl esters and glycosylated derivatives. For instance, compounds **5** and **8** were tentatively characterized as 5-tri-O-protocatechuylglucose and 4-methoxy-cinnamic glycosyl ester with the molecular formulas C_19_H_25_O_9_ and C_27_H_23_O_15_. Both compounds are glycosyl esters of benzoic and cinnamic acids, respectively. These metabolites displayed an [M-H]^−^ at 587.1018 and 397.148 *m*/*z*. Interestingly, there were a few phenolic acid derivatives that only occurred in the SFE extract but not in the maceration extract. This was the case for compounds **6** and **9**, which only occurred in the SFE extract, whereas compound **7** was only detected in the maceration extract.

The stilbenes were the least abundant subclass in both extracts. Two stilbenoid glycosides were tentatively identified in the conventional and SFE extracts. In the former, the stilbenoid glycoside was esterified with gallic acid, and it was detected in positive mode with an [M+H]^+^ at 543.1502 *m*/*z* and a molecular formula of C_27_H_27_O_12_. The latter stilbenoid glycoside was esterified with p-coumaric acid and showed an [M−H]^−^ at 535.1604 *m*/*z* and a molecular formula of C_29_H_27_O_10_. Both molecules were tentatively proposed to be resveratrol 4′-O-β-D-(2″-O-galloyl)-glucopyranoside and resveratrol 4′-O-β-D-(2″-O-cumaroyl)-glucopyranoside. 

A total of 12 flavonoids were tentatively identified in the conventional extract, and 7 of them were also identified in the SFE extract. The subclasses identified in both extracts included flavonoid glycosides, glycosylated chalcones, anthocyanidins, isoflavonoids, and prenylflavonoids. The maceration extract showed a remarkable abundance of these metabolites compared to the SFE extract; however, there were some similarities between both profiles. For example, compounds **2**, **3**, **15**, **17**, and **18**, which correspond to chalconaringenin 2′-rhamnosyl-(1->4)-xyloside, petunidin 3-[6-(rhamnosyl)-2-(xylosyl) glucoside], isoschaftoside, puerarin-6-O-xyloside, and formononetin 7-(2-p-hydroxy benzoyl glucoside) were tentatively identified in both extracts (Table 5). It is important to note that despite observing lower antioxidant capacity values in the SFE extract, its chemical profile also showed a variety of important flavonoid compounds.

Each extraction method’s selectivity in the recovery of flavonoid compounds could be responsible for the subclasses that were only detected in one extract. For example, prenylated flavonoids were uniquely detected in the maceration extract (compounds **19** and **20**), while the glycosylated derivatives of flavanones and flavones were only detected in the SFE extract (compounds **13** and **14**). Moreover, the presence of type 7-O-glycosides (glycosylated flavonoids) was only observed in the conventional extract, which was also the case for the compounds **1**, **11**, and **16**, tentatively characterized as kaempferol 3-arabinofuranoside 7-rhamnofuranoside, phellavin, and amurensin. Surprisingly, type 3-O-glycoside anthocyanins were also identified in both extracts but with a higher abundance in the maceration extract (compounds **3**, **4**, and **12**) (Table 5).

Since the values of the antioxidant capacity of conditions E3, E4, E8, E11, and E12 were higher than the optimized SFE extract, it was assumed that under these conditions (50 °C, 320 bar, 8% *v*/*v* cosolvent; 60 °C, 320 bar, 8%; 60 °C, 235 bar, 10% *v*/*v*; 55 °C, 150 bar, 10% *v*/*v*; 55 °C, 320 bar, 10% *v*/*v*) the recovery of potent antioxidant molecules was not disrupted because it was avoided the competing solubility effect of the scCO_2_ and cosolvent channeling by the vegetal matrix. 

Therefore, it was further investigated whether performing the scCO_2_ extraction under the previous conditions favored the recovery of other potent antioxidant molecules that were not observed in the extract obtained at 60 °C, 320 bar, 10% *v*/*v* cosolvent, and the maceration extract. Table 6 shows the tentatively identified compounds in conditions E3, E4, E8, E11, and E12. As can be observed, eight additional polyphenolic compounds were tentatively identified (compounds **25** to **32**). This result confirmed our hypothesis concerning novel polyphenolic compounds can be obtained using the scCO_2_ extraction, which were not identified in the maceration extracts. In addition, the new phenolic compounds identified in E4, E8, E11, and E12 might be responsible for the high antioxidant capacity in those extracts of scCO_2_ extraction. In fact, compound **30** (Myricetin 3-(2″-galloylrhamnoside) with the molecular weight formula of C_29_H_27_O_16_ was coincidently identified in E3, E4, E8, E11, and E12. For this particular kind of flavonoids, galloyl substituents in flavonoids have not only been related for exert greater antioxidant capacity in other vegetal materials [63], but galloyl substituents in flavonoids are also crucial for exert their biological properties such antidiabetic and anthelmintic [64,65,66]. In this sense, the obtention of special kinds of polyphenolic compounds from agave species with potent biological activities using scCO2 extraction technology would provide a myriad of applications for food and pharmaceutical sectors.

In the experimental run E12 (55 °C, 320 bar, 10% *v*/*v* cosolvent), four different phenolic compounds were tentatively characterized, namely a cinnamic acid glycosyl ester, flavan glycosylated derivatives, and flavonoid 3-O-glycoside (compounds **27**, **28**, **30**, and **31**). The same compounds were detected in E11, except for compound **27**. The presence of two other compounds was observed in E4, which were a new 3-O-glycoside type anthocyanin and one proanthocyanidin (compounds **25** and **32**). In addition, compound 30 was also detected in E4. Runs E3 and E8 also showed some of the additional compounds in their composition; the former included compounds **25**, **27**, and **30**, while the latter included compounds **26** and **30**. Overall, it can be argued that the extraction of polyphenols using 60 °C, 320 bar, 10% *v*/*v* cosolvent was partial and incomplete because more compounds were detected under the extraction conditions E3, E4, E8, and E12. 

Moreover, the recovery of additional polyphenols in these conditions that were not detected in the optimized SFE extract explains the considerable decrease in the antioxidant capacity. At this point, it is important to highlight the selectivity of each extraction method since scCO_2_ extraction allowed for the recovery of molecules that were not recovered in the maceration extract. 

The UPLC-MS approach has been used in the characterization of polyphenols in agave species because it can analyze samples with complex mixtures of secondary metabolites. The number of studies focusing on identifying phenolic compounds in agave leaves is still limited. A few studies have identified and characterized various subclasses of flavonoids from the leaves of different agave species [7,12,67,68]. 

Morreeuw et al. (2021a) [8] confirmed the presence of mono-, di-, and triglycosylated flavonoids of apigenin, quercetin, kaempferol, myricetin, isorhamnetin, and naringenin in *A. lechuguilla*. Furthermore, it has been reported that the occurrence of flavonoid glycosides in agave plants is certainly related to the crassulacean-acid metabolism of these plants [8,69]. Another feature of glycosylated flavonoids is their high hydrophilicity, which is greater than their parental aglycones [70]. The hydrophilic nature of these molecules is granted by the sugar moieties bonded to the aglycone [71]. In agave plants, glycosylated flavonoids preferentially accumulate rather than in aglycones; thus, lower concentrations of flavonoid aglycones have been reported in agave species [8].

Since glycosylated flavonoids have high solubility, these compounds can be efficiently extracted with polar aqueous mixtures [10]. The current study employed aqueous ethanol in both extraction processes to facilitate the recovery of polar molecules such as polyphenols. To our knowledge, this is the first study to identify kaempferol 3-arabinofuranoside 7-rhamnofuranoside (ternoside), myricetin 3-O-arabinopyranoside, apigenin 6-C-arabinoside 8-C-glucoside, (isoschaftoside), and vitexin 6″-O-malonyl 2″-O-xyloside. 

The C-glycosylated flavonoids isolated from different vegetal matrices have interesting biological activities such as hepatoprotective, anti-inflammatory, antiviral, anticancer, and nematocidal activities [72,73]. In terms of antioxidant capacity, various studies have argued that glycosylation diminishes the antioxidant activity of flavonoids, but at the same time, glycosylation significantly improves their solubility and chemical stability, reinforcing their biological potential in vivo [74]. 

In contrast with flavonoid O-glycosides, C-glycosides are more stable than their parent aglycones due to the presence of the covalent C-C bond between the sugar moiety and flavonoid backbone [75]. In vitro and in vivo studies have shown that this chemical feature also helps them to maintain their antioxidant capacity until they are digested [75,76]. Since the antioxidant capacity of flavonoids is crucial to preventing chronic diseases [77], glycosylated flavonoids from the leaves of *A. angustifolia* Haw. could be great alternatives for treating chronic diseases.

Special types of flavonoids were identified in the maceration and SFE extracts of *A. angustifolia* Haw. such as prenylated and galloylated flavonoid derivatives. Both types are produced from distinctive modifications of the biosynthetic pathway of flavonoids in plants [71,78]. In the case of the prenylated flavonoids (compounds **19**, **20**, and **21**), these metabolites have been isolated from different vegetal materials and showed more pronounced biological activities than their non-prenylated parent compounds [79]. The prenyl side chain in the flavonoid backbone provides these molecules with a higher lipophilicity than non-prenylated flavonoids, which changes the affinity of prenylated flavonoids to cell membrane components, making them more selective for cellular targets of pharmacological importance [80]. On the other hand, galloylated flavonoid derivatives have shown more pronounced chelating and scavenging radical activities, which may result in certain biological activities, such as cytotoxic effects against cancer cells [81], making them potential anticancer agents. Overall, the identified polyphenolic compounds in *A. angustifolia* Haw. could be used to develop novel drugs to treat chronic diseases, and they should be further investigated to evaluate their biological potential.

## 3. Materials and Methods

### 3.1. Chemical Reagents

2,2′-Azino-bis(3-ethylbenzothiazoline-6-sulfonic acid) diammonium salt (ABTS, ≥98% HPLC grade), 2,2-diphenyl-1-picrylhydrazyl (DPPH), 2,4,6-tris(2-pyridyl)-*s*-triazine (TPTZ, ≥98% spectrophotometric grade), (±)-6-hydroxy-2,5,7,8-tetramethylchromane-2-carboxylic acid (Trolox, 97%), quercetin (≥95%, HPLC grade), gallic acid (≥97.5%), Folin–Ciocalteu phenol reagent 2N, sodium hydroxide reagent grade (≥98%), sodium acetate trihydrate ReagentPlus^®^ (≥99.0%), ammonium persulfate (≥98%), sodium nitrite ACS reagent (≥97.0%), acetic acid glacial (≥99.7%), iron(III) chloride (reagent grade, 97%), and methanol ACS reagent (≥99%) were purchased from Sigma-Aldrich^®^ (St. Louis, MO, USA). The other reagents used were sodium carbonate anhydrous ACS, aluminum chloride hexahydrate RA (Fermont^®^, Monterrey N.L., Mexico), chlorohydric acid ACS (37.5%), ethanol ACS, acetone ACS, Milli-Q water, acetonitrile LC-MS LiChrosolv^®^ (Merck, Darmstadt, Germany), and methanol LC-MS LiChrosolv^®^ (Merck, Darmstadt, Germany).

### 3.2. Sample Preparation

Fresh leaves of 6.5-year-old *A. angustifolia* Haw. were provided by mezcal producers from southwest Mexico, specifically from the municipality of San Francisco of Sola, Oaxaca (16°29′31.7″ N 96°57′11.7″ W, 1389 m above sea level). The leaves were washed and chopped into small chunks. Then, the chopped leaves were dehydrated in a convection dryer at 50 °C for 24 h. The dried material was milled and refined using a disc mill until a fine powder was obtained. The particle size of the vegetal powder was eventually reduced by employing a number 40 (425 µm) sieve. The fine powder and fiber were individually stored in black bags at room temperature to protect the sample from environmental conditions.

### 3.3. Solvent Extraction

The maceration was performed using a mass/volume ratio of 1:10 [12], where 0.1 g of fine powder was added to a 1.5 mL Eppendorf tube, and 1 mL of fresh solvent (according to the corresponding condition in the mixture design) was added. The tubes were protected from light and kept at room temperature for five days; each extraction was performed in triplicate. Afterward, the tubes were centrifuged at 604× *g* for 5 min. The supernatant was carefully recovered and transferred to a new Eppendorf tube. The resulting crude extracts were vacuum-dried. Individual aqueous extracts were freeze-dried at −40 °C for 24 h. The dried extracts were stored at −20 °C. Each dried extract was redissolved in 50:50 (*v*/*v*) ethanol–water for the chemical determinations.

### 3.4. Supercritical Extraction

The scCO_2_ extraction was performed in a Thar^®^ SFE500 supercritical fluid extractor (Thar, Process, Pittsburgh, PA, USA). Approximately 30 g of fine powder mixed with 18 g of fiber were homogenized and loaded into the stainless-steel extraction vessel of the extractor. Then, extraction was performed using supercritical carbon dioxide at a flow rate of 15 g/min for three hours. The effects of temperature (55–60 °C), pressure (150–320 bar), and cosolvent percentage (5–10% *v*/*v*) were evaluated. Each supercritical extract was carefully recovered and concentrated using vacuum-drying equipment (Buchi-^®^ R-100) at 45 °C and 60 mbar and stored in an amber flask at −20 °C.

### 3.5. Extraction Yield (EY %)

In each extraction process, the yield was calculated as the percentage of the dry weight of concentrated extract over the weight of the dry leaf as described by Equation (1).

(1) $${\;\;\%\;E}_{yield}=\left[\frac{\left(Dry\;weight\;of\;concentrated\;extract\right)}{\left(Dry\;weight\;of\;vegetal\;material\right)}\right]\times 100$$

### 3.6. Total Phenolic Content Analysis

The total phenolic content was determined using the Folin–Ciocalteu reagent using Rover and Brown’s (2013) methodology [82] with slight modifications. Aliquots of the diluted vegetal extract (20 µL) were taken and placed into a 96-well plate with 10% (*v*/*v*) Folin reagent (100 µL) for 5 min in darkness at 30 °C. Afterward, 7.5% (*w*/*w*) Na_2_CO_3_ was added (80 µL). The total phenolic content was calculated according to the gallic acid calibration curve (y = 0.0031x + 0.0265, R^2^ = 0.9960) prepared from standard solutions (5–300 mg/L). All measurements were obtained using a UV–Vis spectrophotometer microplate reader Thermo Scientific^®^ Multiskan GO (Waltham, MA, USA) after 90 min at 765 nm, in triplicate. The results were expressed as mg gallic acid equivalent/g dry leaf (mg GAE/g DL) ± standard deviation according to the gallic acid calibration curve.

### 3.7. Total Flavonoid Content Analysis

The total flavonoid content was determined using the procedure described by Dewanto et al. (2002) [83] with slight modifications. Aliquots of 1.5 mL of the diluted vegetal extract were taken (225 µL) and placed into a 96-well microplate with 5% NaNO_2_ (70 µL) and 10% AlCl_3_*6H_2_O (150 µL) for 5 min. The reaction was stopped by adding 0.5 mL of 1 M NaOH for 10 min in dark conditions. The total flavonoid content was calculated according to the standard calibration curve (y = 0.0002x + 0.046, R^2^ = 0.9961) prepared from standard solutions of quercetin (5–600 µg/mL). All measurements were taken in triplicate at 415 nm using a UV–Vis spectrophotometer microplate reader Thermo Scientific^®^ Multiskan GO (Waltham, MA, USA). The results were expressed as µg quercetin equivalent/g dry leaf (µg QE/g DL) ± standard deviation according to the quercetin calibration curve.

### 3.8. Radical Cation Scavenging Activity

The radical ABTS^•+^ was prepared by following the procedure described by López-Romero et al. (2018) [13]. Prior to each measurement, the absorbance of the working solution of ABTS^•+^ was adjusted to 0.7 ± 0.02 in a UV–Vis spectrophotometer microplate reader Thermo Scientific^®^ Multiskan GO (Waltham, MA, USA). For the analysis, aliquots of the vegetal diluted extract were taken (20 µL) and placed into a 96-well microplate. Then, the sample was reacted with the working ABTS^•+^ solution (180 µL) for 5 min in dark conditions. The antioxidant activity was calculated according to the standard calibration curve (y = 0.1576x + 4.1845, R^2^ = 0.9983) prepared from standard solutions of Trolox (5–400 µmol). The measurements were repeated in triplicate at 734 nm using a UV–Vis spectrophotometer microplate reader (Thermo Scientific^®^ Multiskan GO). The results were expressed as µmol Trolox equivalent/g dry leaf (µmol TE/g DL) ± standard deviation according to the Trolox calibration curve.

### 3.9. DPPH Radical Scavenging Activity

The radical DPPH^•+^ was prepared by following the procedure described by López-Romero et al. (2018) [13]. Before each measurement, the working solution of DPPH^•+^ was adjusted to 0.7 ± 0.02 in a UV–Vis spectrophotometer microplate reader Thermo Scientific^®^ Multiskan GO (Waltham, MA, USA). For the analysis, aliquots of the vegetal diluted extract were taken (20 µL) and placed into a 96-well microplate. Then, the sample was reacted with the DPPH^•+^ working solution (180 µL) for 30 min in dark conditions. The antioxidant activity was calculated according to the standard calibration curve (y = 0.1236 + 9.4846, R^2^ = 0.9943) prepared from standard solutions of Trolox (5–400 µmol). The measurements were repeated in triplicate at 515 nm using a UV–Vis spectrophotometer microplate reader (Thermo Scientific^®^ Multiskan GO). The results were expressed as µmol Trolox equivalent/g dry leaf (µmol TE/g DL) ± standard deviation according to the Trolox calibration curve.

### 3.10. Ferric Ion-Reducing Antioxidant Power (FRAP)

The FRAP assay was performed by following the procedure described by Zhang et al. (2014) [84]. To prepare the FRAP reagent, 300 mmol/L acetate buffer (pH 3.6), 10 mmol/L TPTZ solution, and 20 mmol/L FeCl_3_ were prepared and mixed at room temperature in a 10:1:1 (*v*/*v*/*v*) ratio. The FRAP solution was stable for three hours and prepared fresh for each analysis. For the analysis, aliquots of the diluted vegetal extract (20 µL) were placed into a 96-well microplate with the FRAP working solution (150 µL) at room temperature for 30 min in the dark. The antioxidant activity was calculated according to the standard calibration curve (y = 0.0018x + 0.0146, R^2^ = 0.9949) prepared from standard solutions of Trolox (10–400 µmol). The measurements were repeated in triplicate at 593 nm using a UV–Vis spectrophotometer microplate reader Thermo Scientific^®^ Multiskan GO (Waltham, MA, USA). The results were expressed as µmol Trolox equivalent/g dry leaf (µmol TE/g DL) ± standard deviation according to the Trolox calibration curve.

### 3.11. Identification of Phenolic Compounds

The phenolic compounds present in the obtained extracts under the optimized conditions were identified using an ESI-QTOF Waters^®^ Xevo G2-XS Qtof Mass Spectrometer coupled with an Acquity^®^ Ultra-High-Performance Liquid Chromatography (UPLC) system H-Class (Milford, MA, USA). The phenolic compounds were first separated using an Agilent Technologies Zorbax-SB-C18 (4.6 × 150 µm, 5 µ) chromatographic column (Santa Clara, CA, USA) at 35 °C.

The mobile phases consisted of acidified water with 0.1% (*v*/*v*) formic acid (A) and acetonitrile acidified with 0.1% (*v*/*v*) formic acid (B). The mobile phase flow was kept to 0.6 mL/min. The elution gradient conditions started at 100% of phase A and decreased to 92% in the next 5 min. During the next 10 min, phase A was reduced to 86%. Then, Phase A decreased to 77% for 14 min. Eventually, the elution gradient was reduced to 68% Phase A in the next 18.5 min and slightly reduced to 63% for the next 28 min. Afterward, Phase A decreased to 50% for 30 min. Finally, Phase A increased sharply to 92% for 32 min and returned to 100% in 35 min.

The separated compounds were ionized in a Waters^®^ ESI-Xevo G2-XS Qtof Mass Spectrometer. The mass acquisition was conducted in negative and positive modes by operating in a mass range of 50–2000 *m*/*z*. The operational parameters were set as follows for positive ion mode: capillary voltage, 3.5 kV; cone voltage, 20 kV; temperature, 100 °C; and desolvation temperature, 400 °C. For negative ion mode: capillary voltage, 3.0 kV; cone voltage, 20 kV; temperature, 100 °C; and desolvation temperature, 400 °C. Argon was used as the collision gas with a cone of 50 L/h. Nitrogen was used as the desolvation gas at 700 L/h.

The tentative identification of compounds was performed by calculating the mass error as described in Equation (2). The results were expressed as the mass error of the tentatively identified metabolites and their retention time. The PubChem, ChemSpider, and Metabolomics JP databases were used to obtain the exact mass of the tentatively identified metabolite.

(2) $$∆m=\left[\frac{\left(Theoretical\;exact\;mass-Experimental\;exact\;mass\right)}{\left(Theoretical\;exact\;mass\right)}\right]\times 10^{6}$$

### 3.12. Experimental Design

The maceration was conducted as described in Section 3.3. The solvent proportion was optimized using a simplex–centroid mixture design to increase the phenolic and flavonoid content and antioxidant activity. As shown in Table 7, the simplex–centroid mixture design consisted of 10 fully randomized conditions, of which 3 corresponded to central points. The conditions included individual, binary, and ternary mixtures of acetone (*AcO*), ethanol (*EtOH*), and water (*w*). The proportion of each solvent was evaluated from 0 to 100% (*v*/*v*). The data were analyzed using the Desing Expert 11 trial version (Stat-Ease^®^ Inc., Minneapolis, MN, USA). Before the statistical analysis, each response variable’s data were transformed using the natural logarithm function. To determine the confidence of the model, the lack of fit was calculated by replicating the experimental design. The ANOVA of the model and post hoc Tukey (α = 95%) were used to evaluate statistical differences between conditions.

Meanwhile, the scCO_2_ extraction was conducted as described in Section 3.4. To maximize the phenolic and flavonoid contents as well as the antioxidant activity, the scCO_2_ extraction was optimized using a Box–Behnken design of three independent variables (temperature, pressure, and % cosolvent) with three levels each. As shown in Table 8, the experimental design consisted of 15 conditions, of which 3 corresponded to central points. All the conditions were fully randomized. The data were analyzed using the Design Expert 11 trial version (Stat-Ease^®^ Inc., Minneapolis, MN, USA). Before the statistical analysis, each response variable’s data were transformed using the natural logarithm function. For each variable, an adequate model was adopted. To determine the confidence of the model, the lack of fit was calculated by replicating the experimental design. The ANOVA of the model and post hoc Tukey (α = 95%) were used to evaluate statistical differences between conditions.

### 3.13. Validation of the Experimental Design

The extractions were performed in duplicate following the theoretical conditions to determine the validity of the predicted values. The previously described chemical determinations of the crude extracts obtained using the optimized extraction conditions were performed in triplicate. Based on the calibration curve equation of each determination, the results were expressed as mg gallic acid equivalent/g dried leaf for total phenolic content, µg quercetin equivalent/g dry leaf for total flavonoid content, and µmol Trolox equivalent/g dried leaf for the antioxidant capacity measured by the ABTS, DPPH, and FRAP assays.

The determination of flavonoid and phenolic contents and antioxidant capacity were performed as described in Section 3.6, Section 3.7, Section 3.8, Section 3.9 and Section 3.10. The results were expressed with the standard deviation according to the calibration curve performed for each determination.

## 4. Conclusions

From the outcomes of this study, it can be concluded that the binary aqueous ethanolic solvent mixture (63:37 *v*/*v*) efficiently extracted higher phenolic and flavonoid contents than the individual solvents. In addition, a strong correlation between TPC and TFC with the antioxidant capacity was observed; extracts with a high TPC and TFC obtained at the optimal condition had a high antioxidant capacity. In the scCO_2_ extraction, *A. angustofilia* Haw. vegetal powder may promote swelling, and compaction could create channeling that affects the flow of the cosolvent into the sample matrix.

Using LC-ESI-QTof/MS, new types of glycosylated and prenylated flavonoids were tentatively identified for the first time in *A. angustifolia* Haw. as well as new glycosyl derivatives of hydroxycinnamic and cinnamic acids. In maceration extracts, the antioxidant capacity was attributed to the presence of such compounds. Although there were a few similarities in the polyphenolic compounds identified in the extracts from maceration and supercritical dioxide extractions, the latter extract contained novel flavonoid derivatives, such as myricetin 3-(2″-galloylrhamnoside), myricetin 3-alpha-L-arabinopyranoside, epigallocatechin 3-O-(3,5-di-O-methylgallate), vitexin 6″-O-malonyl 2″-O-xyloside, peonidin 3-rhamnoside-5-glucoside, procyanidin B2, and 3,4-dihydroxy-5-methoxycinnamoyl 6-O-(beta-D-glucopyranosyl)-beta-D-glucopyranoside. To our knowledge, this is the first study to report these polyphenolic compounds in *A. angustifolia* Haw. leaves.

The additional identified polyphenolic compounds were only detected in the extracts obtained using high cosolvent concentrations, indicating that the optimized cosolvent from the maceration process assisted the supercritical carbon dioxide extraction in obtaining novel types of polyphenolic compounds from *A. angustifolia* Haw. leaves. Owing to the biological potential of the polyphenols, further research should be conducted to assess their biological activities and further expand the technological uses of this agro-waste.

## Figures and Tables

**Table 1:** Correlation analysis between the phenolic content and antioxidant capacity of crude extracts from leaves of *Agave angustifolia* Haw. obtained by maceration.

Phenolic Content	*r* ^2^	Flavonoid Content	*r* ^2^
ABTS *	0.610	ABTS *	0.518
DPPH *	0.824	DPPH *	0.775
FRAP *	0.963	FRAP *	0.880
DPPH vs. FRAP *	0.845	-	-

* Indicate statistical differences (*p* < 0.05).

**Table 2:** Experimental validation of the theoretical conditions of maceration extraction predicted by the reduced cubic model.

(63:37% *v*/*v* Water–Ethanol)					
Predicted Model Values ^a^					
TPC	TFC	Yield	ABTS	DPPH	FRAP
mg GAE/g DL	µg QE/g DL	%	AC: µmol TE/g DL		
25.26 ± 3.60	3.86 ± 0.65	30 ± 6	23.57 ± 3.51	15.73 ± 2.56	16.00 ± 2
Experimental values ^b^					
TPC	TFC	Yield (%)	ABTS	DPPH	FRAP
mg GAE/g DL	µg QE/g DL	%	AC: µmol TE/g DL		
27.92 ± 0.90	12.85 ± 0.53	22.20 ± 3.30	32.67 ± 0.91	17.30 ± 0.36	13.92 ± 0.78

^a^ Predicted value ± standard deviation calculated by the model. ^b^ The experimental value is the average of three replicates ± standard deviation. AC: Antioxidant capacity, TPC: Total phenolic content, TFC: Total flavonoid content.

**Table 3:** Correlation analysis of the phenolic and flavonoid content in supercritical extraction.

Phenolic Content	*r* ^2^	Flavonoid Content	*r* ^2^
ABTS *	0.826	ABTS *	0.747
DPPH *	0.926	DPPH *	0.932
FRAP *	0.949	FRAP *	0.902
ABTS vs. DPPH *	0.827		
ABTS vs. FRAP *	0.930		
DPPH vs. FRAP *	0.930	-	-

* indicate significant differences (*p* < 0.05).

**Table 4:** Experimental validation of the theoretical conditions of supercritical carbon dioxide extraction predicted by the quadratic model and special reduced cubic model.

Validation of the Optimized Extraction Conditions (60 °C, 320 bar, 10% *v*/*v*)				
Predicted Values ^a^				
TPC	TFC	ABTS ^RC^	DPPH	FRAP
mg GAE/g DL	µg QE/g DL	AC: µmol TE/g DL		
69 ± 6.00	65 ± 6.00	245 ± 42.00	75.00 ± 5.00	44.60 ± 3.41
Experimental Values ^b^				
TPC	TFC	ABTS	DPPH	FRAP
mg GAE/g DL	µg QE/g DL	AC: µmol TE/g DL		
8.48 ± 1.01	8.35 ± 0.21	11.25 ± 0.57	4.96 ± 0.06	11.37 ± 0.21

^a^ Predicted value ± standard deviation calculated by the model. ^b^ The experimental value is the average of three replicates ± standard deviation. AC: Antioxidant capacity; TPC: Total phenolic content; TFC: Total flavonoid content. ^RC^ Reduced cubic model (predicted values).

**Table 5:** Tentatively identified compounds in leaf extracts of *Agave angustifolia* Haw. obtained by maceration and scCO_2_.

No.	Compound	Molecular Weight Formula	RT (min)	Ionization Mode	Theoretical (*m*/*z*)	Observed (*m*/*z*)	Mass Error	Extraction Method ^a^
**1**	Kaempferol 3-arabinofuranoside 7-rhamnofuranoside	C_26_H_27_O_14_	1.76	[M-H]	563.1401	563.1356	7.99	M
**2**	Chalconaringenin 2′-rhamnosyl-(1->4)-xyloside	C_26_H_29_O_13_	6.76	[M-H]	549.1608	549.1567	7.47	M, SFE
**3**	Petunidin 3-[6-(rhamnosyl)-2-(xylosyl) glucoside]	C_33_H_42_O_20_^+^	11.35	[M+H]	758.2269	758.2251	2.37	M, SFE
**4**	Cyanidin 3-O-rutinoside	C_27_H_32_O_15_^+^	11.48	[M+H]	596.174	596.176	−3.35	M
**5**	Tri-O-protocatechuoylglucose	C_27_H_23_O_15_	9.58	[M-H]	587.1037	587.1018	3.24	M, SFE
**6**	3,4,5-Tri(galloyloxy)benzoic acid	C_28_H_17_O_17_	9.51	[M-H]	625.0465	625.0443	3.52	SFE
**7**	eugenol rutinoside	C_22_H_31_O_11_	2.11	[M-H]	471.1866	471.184	5.52	M
**8**	4-Methoxycinnamic acid (2S)-2-[(beta-d-glucopyranosyl)oxy]propyl ester	C_19_H_25_O_9_	2.03	[M-H]	397.1498	397.148	4.53	M, SFE
**9**	4-[6-O-(2,3-Dihydroxy-2-ethylbutyryl)-beta-d-glucopyranosyloxy]cinnamic acid	C_21_H_27_O_11_	3.019	[M-H]	455.1553	455.1534	4.17	SFE
**10**	Mexoticin 3-′O-(6-O-d-apiofuranosyl-d-glucopyranoside)	C_27_H_37_O_15_	5.1	[M-H]	601.2132	601.2161	−4.82	M
**11**	Phellavin	C_26_H_31_O_12_	1.79	[M-H]	535.1815	535.183	−2.80	M
**12**	Delphinidin-3-fructoside	C_21_H_29_O_12_	2.11	[M-H]	473.1659	473.1684	−5.28	M
**13**	7-Hydroxy-7″-(D-glucopyranosyloxy)-3,8″-bi [4′,5-dihydroxyflavanone]	C_36_H_32_O_15_	4.46	[M+H]	705.1819	705.1823	−0.57	SFE
**14**	5,4′-Dihidroxy-7,8,2′,3′-tetramethoxy flavone 5-glucoside	C_25_H_27_O_13_	2.19	[M-H]	535.1451	535.1451	0.00	SFE
**15**	Isoschaftoside	C_26_H_27_O_14_	1.85	[M-H]	563.1401	563.1404	−0.53	M, SFE
**16**	Amurensin	C_26_H_29_O_12_	9.76	[M-H]	533.1659	533.1624	6.56	M
**17**	Puerarin-6-O-xyloside	C_26_H_27_O_13_	1.71	[M-H]	547.1451	547.1433	3.29	M, SFE
**18**	Formononetin 7-(2-p-hydroxybenzoylglucoside)	C_29_H_25_O_11_	9.48	[M-H]	549.1397	549.1376	3.82	M, SFE
**19**	5,4′-Dihydroxy-6-C-prenylflavanone 4′-xylosyl-(1->2)-rhamnoside	C_31_H_37_O_12_	5.32	[M-H]	601.2285	601.2261	3.99	M
**20**	3,5,8,3′,4′-Pentamethoxy-7-prenyloxyflavone	C_25_H_27_O_8_	2.12	[M-H]	455.1706	455.1708	−0.44	M
**21**	Resveratrol 4′-O-β-D-(2″-O-galloyl)-glucopyranoside	C_27_H_27_O_12_	5.43	[M+H]	543.1502	543.153	−5.16	M
**22**	Resveratrol 4′-O-β-D-(2″-O-cumaroyl)-glucopyranoside	C_29_H_27_O_10_	2.07	[M-H]	535.1604	535.1594	1.87	SFE
**23**	Lippioside II	C_25_H_29_O_14_	2.07	[M-H]	553.1557	553.1513	7.95	M
**24**	Muraxanthone	C_26_H_23_O_13_	1.86	[M+H]	543.1138	543.1101	6.81	M

^a^ M: maceration extraction; SFE: supercritical fluid extraction.

**Table 6:** Tentatively identified compounds in leaf extracts of *Agave angustifolia* Haw. obtained by scCO_2_ extraction.

No.	Compound	Molecular Weight Formula	RT (min)	Ionization Mode	Theoretical (*m*/*z*)	Observed (*m*/*z*)	Mass Error (ppm)	Treatment ^a^
**25**	Peonidin 3-rhamnoside-5-glucoside *	C_28_H_34_O_15_^+^	9.27	[M+H]	610.1897	610.1878	3.11	E3, E4
**4**	Cyanidin 3-O-rutinoside	C_27_H_32_O_15_^+^	11.8	[M+H]	596.174	596.176	−3.35	E3, E4, E8, E11, E12
**3**	Petunidin 3-[6 (rhamnosyl)-2-(xylosyl) glucoside]	C_33_H_42_O_20_^+^	11.33	[M+H]	758.2269	758.2251	2.37	E3, E8, E11, E12
**5**	Tri-O-protocatechuoylglucose	C_27_H_23_O_15_	2.58	[M-H]	587.1037	587.1018	3.24	E3, E11, E12
**26**	3,4-Dihydroxychalcone 4-beta-L-arabinopyranosyl-(1->4)-galactoside *	C_26_H_29_O_12_	3.16	[M-H]	533.1659	533.1624	6.56	E8
**2**	Chalconaringenin 2′-rhamnosyl-(1->4) -xyloside	C_26_H_29_O_13_	2.27	[M-H]	549.1608	549.1616	−1.46	E4
**27**	3,4-Dihydroxy-5-methoxycinnamoyl 6-O-(beta-D-glucopyranosyl)-beta-D-glucopyranoside *	C_22_H_29_O_15_	2.2	[M-H]	533.1506	533.1529	−4.31	E3, E12
**8**	4-Methoxycinnamic acid (2S)-2-[(beta-D-glucopyranosyl)oxy]propyl ester	C_19_H_25_O_9_	2.15	[M-H]	397.1498	397.148	4.53	E8
**13**	7-Hydroxy-7″-(D-glucopyranosyloxy)-3,8″-bi [4′,5-dihydroxyflavanone]	C_36_H_33_O_15_	6.76	[M+H]	705.1819	705.1823	−0.57	E3, E4, E8, E11, E12
**28**	Epigallocatechin 3-O-(3,5-di-O-methylgallate) *	C_24_H_21_O_11_	11.98	[M-H]	485.1084	485.1081	0.62	E3, E11, E12
**1**	Kaempferol 3-arabinofuranoside 7-rhamnofuranoside	C_26_H_27_O_14_	1.82	[M-H]	563.1401	563.1356	7.99	E8
**15**	Isoschaftoside	C_26_H_27_O_14_	2	[M-H]	563.1401	563.1404	−0.53	E8
**29**	Vitexin 6″-O-malonyl 2″-O-xyloside *	C_29_H_31_O_17_	2.93	[M+H]	651.156	651.1562	−0.31	E4
**30**	Myricetin 3-(2″-galloylrhamnoside) *	C_29_H_27_O_16_	4.77	[M+H]	631.1298	631.1301	−0.48	E3, E4, E8, E11, E12
**31**	Myricetin 3-alpha-L-arabinopyranoside *	C_20_H_17_O_12_	1.71	[M-H]	449.072	449.0713	1.56	E11, E12
**18**	Formononetin 7-O-(2″-p-hydroxybenzoylglucoside)	C_29_H_25_O_11_	9.28	[M-H]	549.1397	549.1376	3.82	E3, E8, E11, E12
**32**	Procyanidin B2 *	C_30_H_25_O_12_	2.37	[M-H]	577.1346	577.1376	−5.20	E4
**21**	(Pieceid-2″-O-gallate)	C_27_H_27_O_12_	5.43	[M+H]	543.1502	543.1483	3.50	E3, E4, E8, E11, E12

* Correspond to new polyphenolic compounds tentatively identified in the supercritical extracts. ^a^ E3 (50 °C, 320 bar, 8% *v*/*v* cosolvent), E4 (60 °C, 320 bar, 8% *v*/*v* cosolvent), E8 (60 °C, 235 bar, 10% *v*/*v* cosolvent), E11 (55 °C, 150 bar, 10% *v*/*v* cosolvent), E12 ((55 °C, 320 bar, 10% *v*/*v* cosolvent).

**Table 7:** Experimental conditions of the simplex–centroid mixture design to optimize the maceration extraction.

Treatment	Solvent Mixture		
	Acetone (% *v*/*v*)	Ethanol (% *v*/*v*)	Water (% *v*/*v*)
E1	100	0	0
E2	0	100	0
E3	0	0	100
E4	50	50	0
E5	50	0	50
E6	0	50	50
E7	33.33	33.33	33.33
E8	66.67	16.67	16.67
E9	16.67	66.67	16.67
E10	16.67	16.67	66.67

**Table 8:** Experimental conditions of the Box–Behnken design to optimize the scCO_2_ extraction.

Treatment	Temperature (°C)	Pressure (Bar)	Modifier (% *v*/*v*)
E1	50	150	8
E2	60	150	8
E3	50	320	8
E4	60	320	8
E5	50	235	5
E6	60	235	5
E7	50	235	10
E8	60	235	10
E9	55	150	5
E10	55	320	5
E11	55	150	10
E12	55	320	10
E13	55	235	8
E14	55	235	8
E15	55	235	8

## Data Availability

All data are contained within the article.

## Supplementary Materials

The following supporting information can be downloaded at https://www.mdpi.com/article/10.3390/molecules29051137/s1, Figure S1: Desirability function and predicted values of the reduced cubic model in the maceration extraction process, Figure S2: Correlation between the predicted responses by the reduced cubic model in the optimization of the maceration process using a simplex-centroid design vs. the observed values of each response variable; Table S1: Analysis of variance and model fitting of phenolic and flavonoid content evaluated in the maceration extraction, Table S2: Analysis of variance and model fitting of the antioxidant activity evaluated by using the ABTS^•+^, DPPH^•+^, and FRAP assays in the maceration extraction, Table S3: Analysis of variance and model fitting of phenolic and flavonoid content evaluated in the scCO_2_ extraction, and Table S4: Analysis of variance and model fitting of the antioxidant activity evaluated by using the ABTS^•+^, DPPH^•+^, and FRAP assays in the scCO_2_ extraction.
