# Supplementary material for: Agave angustifolia Haw. Leaves as a Potential Source of Bioactive Compounds: Extraction Optimization and Extract Characterization
Source: Molecules. 2024 Mar 3;29(5):1137. doi: 10.3390/molecules29051137 (PMC10935067; doi:10.3390/molecules29051137)
Supplement: Supplementary file 1 [file molecules-29-01137-s001.zip › molecules-2829327-supplementary.pdf]

Supplementary Table S1. *Analysis of variance and model fitting of phenolic and flavonoid content evaluated in the maceration extraction.*

Phenolic content				Flavonoid content				Total extraction yield			
Statistical data	Regression coefficient	F-value	p-value	Statistical data	Regression coefficient	F-value	p-value	Statistical data	Regression coefficient	F-value	p-value
Model	-	201.19	<0.0001	Model	-	35.47	<0.0001	Model		24.19	<0.0001
Linear mixture	-	516.05	<0.0001	Linear Mixture	-	73.42	<0.0001	Linear Mixture		44.12	<0.0001
*A	-0.1802		<	A	-0.1640			A	+3.01		
*B	+1.44			B	+0.5832			B	+2.51		
*C	+2.82			C	+1.04			C	+3.13		
AB	+2.36	34.57	<0.0001	AB	+0.5024	1.10	0.3065	AB	-2.01	17.95	0.0004
AC	+7.88	386.90	<0.0001	AC	+4.51	88.53	<0.0001	AC	+1.83	14.91	0.0009
BC	+3.91	95.20	<0.0001	BC	+1.98	17.12	0.0005	BC	+2.08	19.15	0.0003
ABC	-4.38	2.78	0.1104	ABC	-0.0645	0.0004	0.9838	ABC	+5.11	2.69	0.1156
AB(A-B)	-3.78	8.08	0.0098	AB(A-B)	-6.16	14.98	0.0009	AB(A-B)	-5.14	10.63	0.0037
AC(A-C)	+5.96	20.06	0.0002	AC(A-C)	+3.13	3.87	0.0625	AC(A-C)	-2.89	3.36	0.0809
Lack of fit			0.3733	Lack of fit			0.8612	Lack of fit			0.2241
**R^2	0.9871			R^2	0.9311			R^2	0.9021		
***Adj R^2	0.9822			Adj R^2	0.9048			Adj R^2	0.8648		
Predicted R^2	0.9741			Predicted R^2	0.8619			Predicted R^2	0.8237		
Adequate precision	44.63			Adequate precision	18.62			Adequate precision	15.01		

*The letters A, B, and C indicated the individual solvents acetone, ethanol and water, respectively. The combination of these letters represents the mixture of the solvent system.

**R^2: Coefficient of determination.

***Adj R^2: Adjusted coefficient of determination.

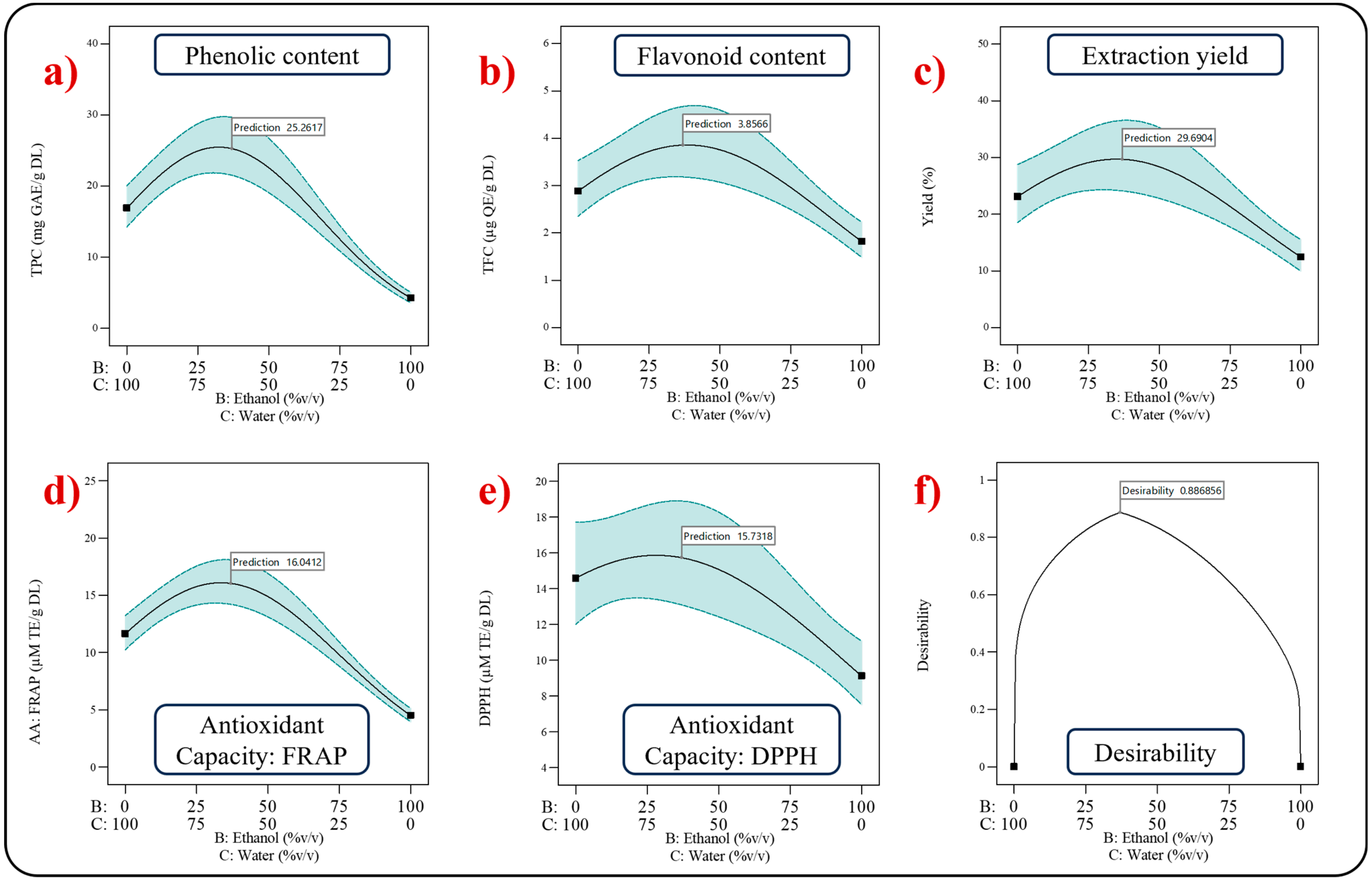

Figure S1. Desirability function and predicted values of the reduced cubic model in the maceration extraction process a) phenolic content, b) flavonoid content, c) extraction yield, d) antioxidant capacity FRAP, e) antioxidant capacity DPPH, f) desirability function of the model.

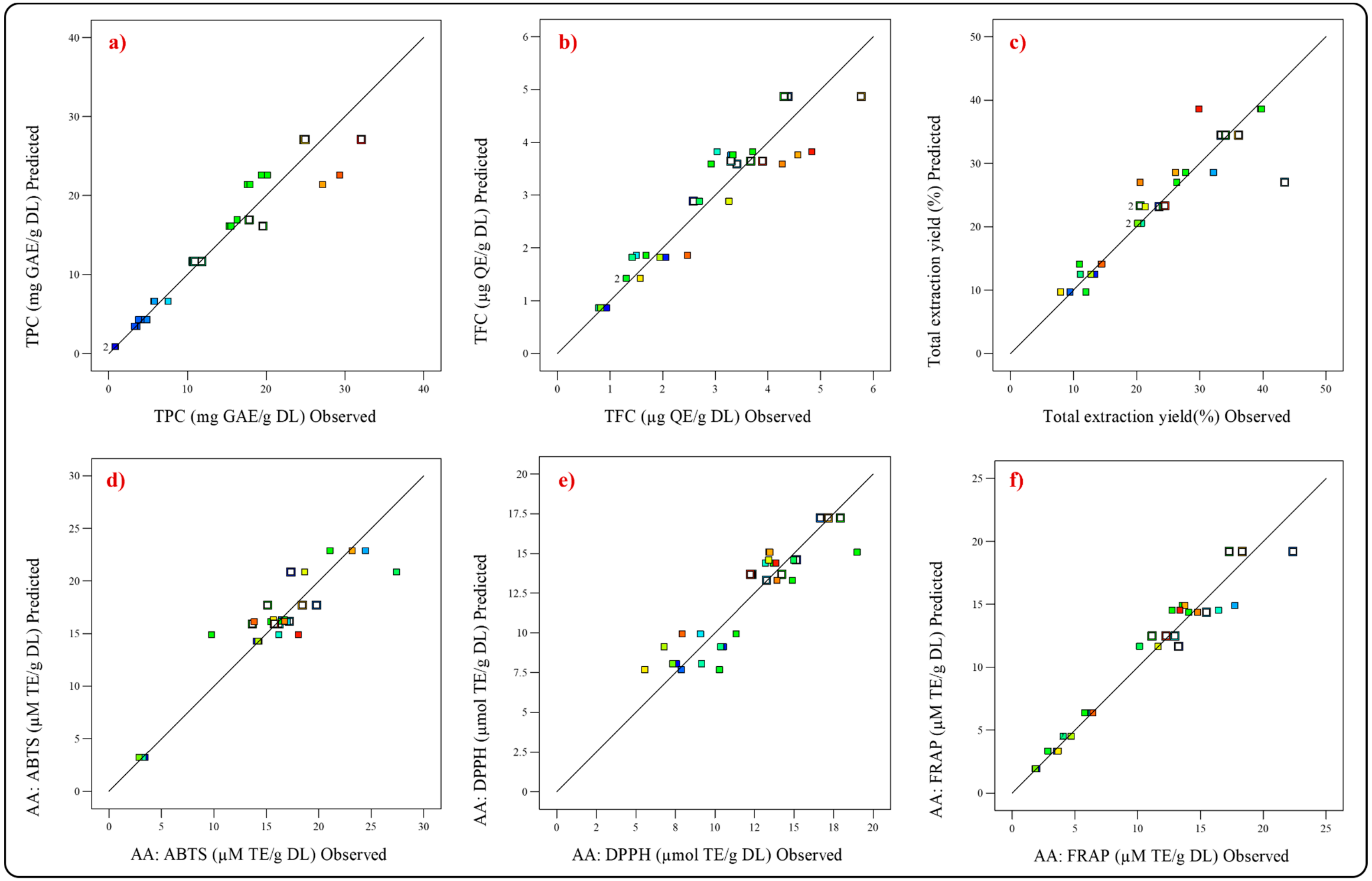

Figure S2. Correlation between the predicted responses by the reduced cubic model in the optimization of the maceration process using a simplex-centroid design vs. the observed values of each response variable a) phenolic content, b) flavonoid content, c) extraction yield, antioxidant capacity: ABTS (d); DPPH (e) and FRAP (f).

Supplementary Table S2. *Analysis of variance and model fitting of the antioxidant activity evaluated by using the ABTS•+, DPPH•+, and FRAP assays in the maceration extraction.*

**AA: ABTS[**]				**AA: DPPH**				**AA: FRAP**			
Statistical data	Regression coefficient	F-value	p-value	Statistical data	Regression coefficient	F-value	p-value	Statistical data	Regression coefficient	F-value	p-value
Model	-	46.05	<0.0001	Model		10.74	<0.0001	Model		176.01	<0.0001
Linear mixture	-	102.51	<0.0001	Linear Mixture		26.75	<0.0001	Linear Mixture		394.89	<0.0001
[*]A	+1.15			A	+2.07			A	+0.6463		
[*]B	+2.65			B	+2.20			B	+1.50		
[*]C	+3.03			C	+2.67			C	+2.45		
AB	+3.51	70.40	<0.0001	AB	-0.4398	0.9219	0.3479	AB	+0.4959	2.70	0.1152
AC	+3.09	54.32	<0.0001	AC	+1.86	16.47	0.0006	AC	+5.60	344.79	<0.0001
BC	+1.13	7.28	0.0135	BC	+1.07	5.47	0.0293	BC	+2.88	91.40	<0.0001
ABC	-9.84	12.84	0.0018	ABC	-0.3571	0.0141	0.9065	ABC	+3.48	3.08	0.0937
AB(A-B)	+2.26	2.64	0.1192	AB(A-B)	-4.20	7.62	0.0117	AB(A-B)	-7.32	53.35	<0.0001
AC(A-C)	+6.23	20.04	0.0002	AC(A-C)	+2.57	2.85	0.1059	AC(A-C)	+6.14	37.57	<0.0001
Lack of fit			0.9262	Lack of fit			0.2734	Lack of fit			0.3827
[***]R^2	0.9461			R^2	0.8035			R^2	0.9853		
[****]Adj R^2	0.9255			Adj R^2	0.7287			Adj R^2	0.9797		
Predicted R^2	0.8906			**Predicted** R^2	0.5798			**Predicted** R^2	0.9689		
Adequate precision	24.19			Adequate precision	9.11			Adequate precision	39.32		

*The letters A, B, and C indicated the individual solvents acetone, ethanol, and water, respectively. The combination of these letters represents the mixture of the solvent system.

**AA: Antioxidant activity.

***R^2: Coefficient of determination.

****Adj R^2: Adjusted coefficient of determination.

Supplementary Table S3. *Analysis of variance and model fitting of phenolic and flavonoid content evaluated in the $scCO_2$ extraction.*

**Phenolic content**				**Flavonoid content**			
Statistical data	Regression coefficient	F-value	p-value	Statistical data	Regression coefficient	F-value	p-value
Model	+22.21	39.87	<0.0001	Model	+19.97	39.98	<0.0001
A: Temperature	+4.22	10.17	0.0031	A: Temperature	+8.03	49.32	<0.0001
B: Pressure	+6.95	27.65	<0.0001	B: Pressure	+6.45	31.87	<0.0001
C: Modifier	+19.51	222.08	<0.0001	C: Modifier	+16.01	200.04	<0.0001
*AB	+0.9770	0.2785	0.6012	AB	-1.19	0.5513	0.4631
*AC	+5.88	10.28	0.0030	AC	+5.13	10.48	0.0028
*BC	+7.50	16.72	0.0003	BC	+7.77	24.03	<0.0001
A^2	-2.03	1.11	0.3005	A^2	-3.55	4.53	0.0408
B^2	+10.23	28.16	<0.0001	B^2	+6.54	15.38	0.0004
C^2	-6.45	10.15	0.0032	C^2	-0.5631	0.1033	0.7499
Lack of fit			0.6740	Lack of fit			0.1338
**R^2	0.9158			R^2	0.9160		
Predicted R^2	0.8425			Predicted R^2	0.8411		
***Adj R^2	0.8928			Adj R^2	0.8931		
Adequate precision	20.48			Adequate precision	20.86		

*The combination of these letters represents the interaction between the independent variables evaluated in the experimental design.

**R^2: Coefficient of determination.

***Adj R^2: Adjusted coefficient of determination.

Supplementary Table S4. *Analysis of variance and model fitting of the antioxidant activity evaluated by using the ABTS*$^{\bullet+}$*, DPPH*$^{\bullet+}$*, and FRAP assays in the* $scCO_2$ *extraction.*

[**]AA: ABTS				AA: DPPH				AA: FRAP			
Statistical data	Regression coefficient	F-value	p-value	Statistical data	Regression coefficient	F-value	p-value	Statistical data	Regression coefficient	F-value	p-value
Model	+197.15	33.26	<0.0001	Model	+30.91	87.02	<0.0001	Model	+15.39	63.28	<0.0001
A: Temperature	+51.15	34.55	<0.0001	A: Temperature	+7.85	68.75	<0.0001	A: Temperature	+1.95	7.66	0.0092
B: Pressure	+29.64	5.91	0.0208	B: Pressure	+6.87	52.65	<0.0001	B: Pressure	+4.83	47.13	<0.0001
C: Modifier	+94.46	120.16	<0.0001	C: Modifier	+20.46	476.53	<0.0001	C: Modifier	+14.48	432.63	<0.0001
[*]AB	-39.76	10.65	0.0026	AB	+1.34	1.02	0.3190	AB	-0.1710	0.0302	0.8632
[*]AC	+41.92	12.07	0.0015	AC	+5.26	16.09	0.0003	AC	+2.44	6.26	0.0175
[*]BC	+28.18	5.35	0.0273	BC	+5.99	20.84	<0.0001	BC	+5.39	30.63	<0.0001
A^2	-34.67	7.47	0.0101	A^2	-2.76	4.02	0.0533	A^2	-1.44	1.96	0.1705
B^2	+55.47	19.13	0.0001	B^2	+7.77	31.76	<0.0001	B^2	+3.16	9.51	0.0041
C^2	-111.33	69.71	<0.0001	C^2	-8.75	36.37	<0.0001	C^2	-1.42	1.74	0.1960
A^2B	-67.18	14.90	0.0005	-	-	-	-	-	-	-	-
Lack of fit			0.0981	Lack of fit			0.5328	Lack of fit			0.9225
[***]R^2	0.9122			R^2	0.9596			R^2	0.9452		
Predicted R^2	0.8173			Predicted R^2	0.9234			Predicted R^2	0.9053		
[****]Adj R^2	0.8848			Adj R^2	0.9485			Adj R^2	0.9303		
Adequate precision	18.71			Adequate precision	28.55			Adequate precision	25.46		

*The combination of these letters represents the interaction between the independent variables evaluated in the experimental design.

**AA: Antioxidant activity.

***R^2: Coefficient of determination.

****Adj R^2: Adjusted coefficient of determination.
